# A novel affinity-based method for the isolation of highly purified extracellular vesicles

**DOI:** 10.1038/srep33935

**Published:** 2016-09-23

**Authors:** Wataru Nakai, Takeshi Yoshida, Diego Diez, Yuji Miyatake, Takahiro Nishibu, Naoko Imawaka, Ken Naruse, Yoshifusa Sadamura, Rikinari Hanayama

**Affiliations:** 1 Laboratory of Immune Network, WPI Immunology Frontier Research Center (IFReC), Osaka University, 3-1 Yamada-oka, Suita, Osaka 565-0871, Japan.; 2 Department of Immunology, Kanazawa University Graduate School of Medical Sciences, 13-1 Takara, Kanazawa, Ishikawa 920-8640, Japan; 3 Quantitative Immunology Research Unit, WPI Immunology Frontier Research Center (IFReC), Osaka University, 3-1 Yamada-oka, Suita, Osaka 565-0871, Japan; 4 Life Science Research Laboratories, Wako Pure Chemical Industries Ltd., 6-1 Takada-Cho, Amagasaki, Hyogo 661-0963, Japan; 5 PRESTO, Japan Science and Technology Agency (JST), 4-1-8 Honcho, Kawaguchi, Saitama 332-0012, Japan

## Abstract

Extracellular vesicles (EVs) such as exosomes and microvesicles serve as messengers of intercellular network, allowing exchange of cellular components between cells. EVs carry lipids, proteins, and RNAs derived from their producing cells, and have potential as biomarkers specific to cell types and even cellular states. However, conventional methods (such as ultracentrifugation or polymeric precipitation) for isolating EVs have disadvantages regarding purity and feasibility. Here, we have developed a novel method for EV purification by using Tim4 protein, which specifically binds the phosphatidylserine displayed on the surface of EVs. Because the binding is Ca^2+^-dependent, intact EVs can be easily released from Tim4 by adding Ca^2+^ chelators. Tim4 purification, which we have applied to cell conditioned media and biofluids, is capable of yielding EVs of a higher purity than those obtained using conventional methods. The lower contamination found in Tim4-purified EV preparations allows more EV-specific proteins to be detected by mass spectrometry, enabling better characterization and quantification of different EV populations’ proteomes. Tim4 protein can also be used as a powerful tool for quantification of EVs in both ELISA and flow cytometry formats. Thus, the affinity of Tim4 for EVs will find abundant applications in EV studies.

Extracellular vesicles (EVs) are small membrane vesicles, composed of a lipid bilayer with inserted transmembrane proteins, enclosing cytosolic components (protein, mRNA, and miRNA) derived from the EV-producing cells^1^,^2^. EVs consist of a heterogeneous population of exosomes (30–100 nm in diameter) of endocytic origin and microvesicles (100–1000 nm in diameter), shed fragments of plasma membrane. Recent studies have shown that EVs act as messengers of intercellular communication networks, allowing cells to transfer lipids, proteins, and RNAs between the producing cells and target cells^3^,^4^. The contents of EVs may potentially be useful as cell-type specific biomarkers^5^,^6^,^7^,^8^. Therefore, detailed characterization of EV components could be very important for developing novel strategies against various diseases such as cancer, neurodegenerative diseases, and immune disorders.

Although there has been an accelerating increase in studies of EV components from various cell culture supernatants or biofluids such as blood, urine, or saliva, fundamental problems still remain to be solved regarding the purity of collected EVs and the efficiency of the purification methods. As cells release heterogeneous EVs, which can be classified by size (such as small EVs (sEVs) and large EVs), sequential centrifugation is widely used to enrich certain subtypes of EVs^9^. In general, cell conditioned media or biofluids are differentially centrifuged at 300× *g*, 2,000× *g*, and 10,000× *g* to remove cells, cellular debris, and large EVs, and the supernatants are filtered through 220-nm pore filters to enrich sEVs (10K sup), which mainly contain exosomes. Conventional methods to isolate sEVs from the 10K sup are based on ultracentrifugation (UC), polymer-based precipitation, or immunoprecipitation^10^. Among them, UC is probably the most commonly used method to purify sEVs. However, UC requires an expensive instrument with limited capacity for parallel processing. Additionally, the amount of collected sEVs is not consistent due to small and fragile pellets or sedimentation efficiency. An alternative method, gaining popularity because of its simplicity and feasibility, is polymer-based precipitation such as polyethylene glycol (PEG) precipitation. Although this method efficiently recovers sEVs, a large proportion of non-sEV contaminants are also precipitated at the same time. These contaminants interfere with subsequent analyses such as lowering of the number of detectable peptides during mass spectrometry. Moreover, it is hard to distinguish whether the biological functions found associated with preparations of sEVs are truly mediated through sEV components or the non-sEV contaminants. Affinity purification by using antibodies against exosome-specific surface proteins such as CD9, CD63, and CD81, or by using EV-binding molecules such as heparin and heat shock protein-binding peptides is another widely used method to isolate sEVs^11^,^12^. However, these methods cannot be used for analyzing the functions of intact sEVs, as it is hard to detach the bound sEVs from the antibodies or EV-binding molecules by using only mild elution conditions. Because of these problems, developing a feasible method to isolate highly purified and intact sEVs has been long awaited.

Here, we report the development of a novel affinity-based method for the isolation of highly purified sEVs by using T-cell immunoglobulin domain and mucin domain-containing protein 4 (Tim4). Tim4 is a type I transmembrane protein expressed on macrophages, which strongly binds phosphatidylserine, displayed not only on apoptotic cells but also on exosomes and microvesicles^13^,^14^,^15^. We employed the extracellular region of Tim4 immobilized on magnetic beads to capture sEVs from various pre-cleared cell conditioned media and biofluids. As Tim4-binding to phosphatidylserine is Ca^2+^-dependent, intact sEVs can be easily released by adding a buffer containing a chelating agent. Most notably, sEVs isolated by our Tim4-affinity purification method were much purer than those isolated using conventional methods, containing fewer non-sEV contaminants as revealed by mass spectrometry. Capturing EVs with Tim4 protein was also used for isolation of large EVs from 10,000× *g* pellet of cell conditioned media (10K pellet) and sensitive quantification of EVs by ELISA and flow cytometry. These results establish the usefulness of purification via Tim4 protein in characterization of the *bona fide* functions of EVs.

## Results

### Tim4 is a receptor for sEVs

**Figure 1:**
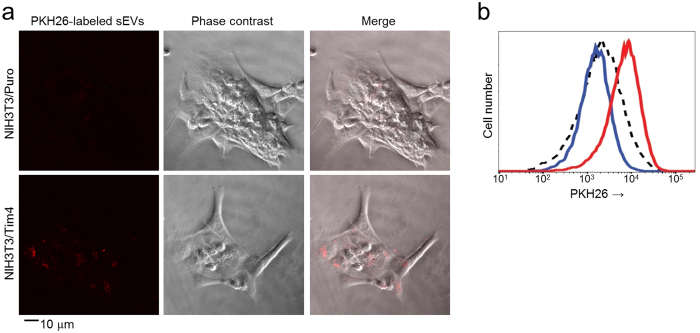
Tim4 captures sEVs. (**a**) K562 cells (5 × 10^4^ cells) were cultured in RPMI 1640 containing 10% EV-depleted FBS and stimulated with 10 μM monensin for 24 h to facilitate the secretion of EVs. The sEVs in 10K sup of K562 cells were isolated by ultracentrifugation and labeled with PKH26 Red Fluorescent Cell Linker Kit (Sigma-Aldrich) according to the manufacturer’s protocol. NIH3T3 cells that expressed a control vector (NIH3T3/Puro) or mouse Tim4 (NIH3T3/Tim4) were cultured with the PKH26-labeled sEVs (red) for 1 h, and then observed by confocal microscopy. Scale bar, 10 μm. (b) The intensities of NIH3T3/Puro (dot line) or NIH3T3/Tim4 (red line) that had been co-cultured with PKH26-labeled sEVs were compared using flow cytometry. NIH3T3/Tim4 cultured with the sEVs in the presence of recombinant D89E mutant of MFG-E8 (10 μg/ml) is shown in blue line.

EVs such as exosomes and microvesicles display phosphatidylserine on their surfaces, allowing recognition by phosphatidylserine receptors such as Tim4^14^,^15^,^16^,^17^. We first examined the ability of Tim4 to capture EVs. As previously reported^18^, stimulation of K562 human erythromyeloblastoid leukemia cell lines with monensin, an ionophore that increases cytosolic free Ca^2+^ concentration, induced secretion of EVs from the cells. We therefore pre-cleared K562 cell conditioned medium by sequential centrifugation at 300× *g*, 2,000× *g*, and 10,000× *g* and also by filtration through 220-nm pore filters to remove cells, cellular debris, and large EVs. We then isolated sEVs from the 10K sup by UC and subsequently labeled them with PKH26 fluorescent dye. When NIH3T3 cells, which do not express Tim4, were co-cultured with the PKH26-labeled sEVs, only a low level of sEVs were internalized in the cells, but expression of Tim4 in the cells strongly augmented the internalization of sEVs (Fig. 1a,b). However, the addition of a high amount (10 μg/ml) of recombinant D89E mutant of MFG-E8 (also known as lactadherin), which binds phosphatidylserine and prevents interaction of phosphatidylserine-binding proteins^19^, to the culture medium abolished the Tim4-mediated augmentation (Fig. 1b). These results demonstrated that Tim4 has an ability to capture sEVs by recognizing phosphatidylserine on their surfaces.

### A novel method to purify sEVs with Tim4

**Figure 2:**
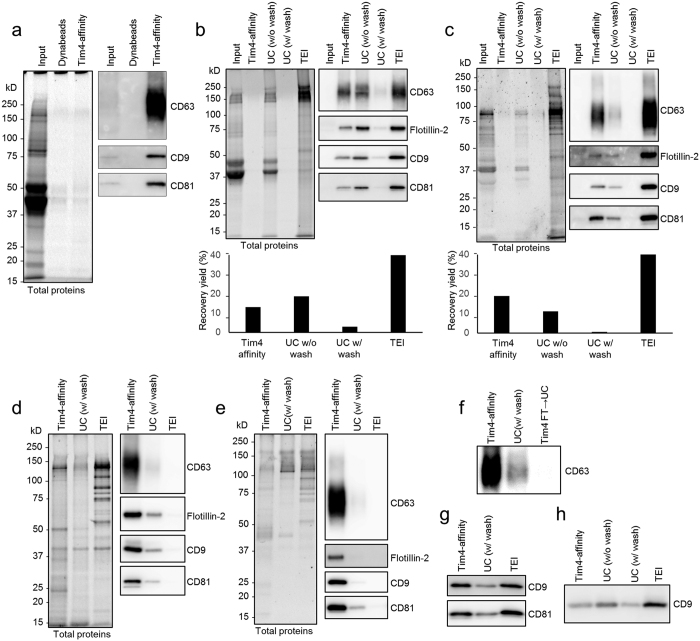
Tim4-affinity purification of sEVs. (**a**) Thioglycollate-elicited mouse peritoneal macrophages (pMac) (3 × 10^5^ cells/ml) were cultured in 10 ml of DMEM containing 5% EV-depleted FBS for 48 h. 10K sup of pMac (4 ml each) were incubated with Dynabeads or mouse Tim4-Fc-conjugated Dynabeads overnight. Bound sEVs were eluted, subjected to SDS-PAGE and analyzed by western blot (right panel) with anti-CD63, anti-CD9, or anti-CD81 antibody. Total proteins in the sEV fractions were visualized by Oriole fluorescent gel stain (left panel). The amount loaded in the input lane was equivalent to 1/1000 volume of the total supernatant. (**b**,**c**) sEVs were purified from 10K sup (4 ml of medium containing 5% EV-depleted FBS) of K562 cells (1 × 10^6^ cells/ml) (**b**) or pMac (4 × 10^5^ cells/ml) (**c**) by each method. The purified sEV fractions were adjusted to the same volume (60 μl), subjected to SDS-PAGE, and analyzed by Oriole stain (left panels) or western blot (right panels). TEI: Total Exosome Isolation. The number of sEV particles in the purified fractions were also analyzed by NTA assay using NanoSight LM10 (Malvern), and the calculated purification recovery yield relative to total sEV particles was plotted in the lower graphs. (**d**,**e**) Protein concentration of purified sEV fractions from K562 cells (**d**) or pMac (**e**) was determined by BCA protein assay, and adjusted to the same in each method. Equal amount of proteins (100 ng) were subjected to SDS-PAGE, and analyzed by Oriole stain (left panels) or western blot (right panels). (**f**) The flow through of 10K sup of K562 cells after Tim4-affinity purification was further ultracentrifuged, and the same proportion of the protein amount to the total purified protein amount as Tim4-affinity purification was loaded to detect residual exosomes by western blot with anti-CD63 antibody (Tim4 FT → UC). (**g**,**h**) sEVs were isolated from 50 μl of mouse serum (**g**) or 500 μl of human urine (**h**) by each method, adjusted to the same volume and analyzed by western blot.

As Tim4 binds phosphatidylserine via an extracellular IgV-like domain in a Ca^2+^-dependent manner^14^, we considered the possibility that the extracellular domain of Tim4 would be useful for the purification of sEVs. We therefore used Tim4-Fc protein in which extracellular region of mouse Tim4 was fused to the Fc region of human IgG^14^. We then conjugated the Tim4-Fc protein with magnetic beads, and examined its ability to purify sEVs, supposing that Tim4-Fc-conjugated beads can capture sEVs in the presence of Ca^2+^ and the bound sEVs can be released from the beads by the addition of elution buffer containing the chelating agent EDTA. However, it is known that sEVs secreted from some innate immune cells (such as immature dendritic cells and thioglycollate-elicited mouse peritoneal macrophages, pMac) or mammary epithelial cells during involution are coated by MFG-E8, which masks the phosphatidylserine^20^,^21^. Since affinity of MFG-E8 to phosphatidylserine is comparable to that of Tim4^14^,^19^ and as shown in Fig. 1b, a high amount of recombinant D89E mutant of MFG-E8 inhibited Tim4-mediated internalization of sEVs, we wondered whether the presence of endogenous MFG-E8 on sEVs might interfere with Tim4 binding to the sEVs. Nevertheless, Tim4-Fc-conjugated beads were still able to isolate sEVs efficiently from 10K sup of pMac which abundantly express MFG-E8, as revealed by western blot analysis with exosomal markers such as CD63, CD9, and CD81 (Fig. 2a).

We next examined the yield and purity of sEVs isolated by our Tim4-affinity method, comparing the efficiency with conventional methods: two UC methods (UC without phosphate buffered saline (PBS) wash, and UC with PBS wash) and a polymer-based precipitation method using a Total Exosome Isolation (TEI) reagent. For these experiments, sEVs were prepared either from K562 cells or pMac by culturing in medium containing 5% FBS which had been deprived of any EVs and potential precipitates in advance. Then, sEVs were isolated from the 10K sup by each method, and the isolated sEVs were examined by western blot analysis against exosomal markers and also by Nanoparticle Tracking Analysis (NTA) using the NanoSight apparatus^22^. The TEI method could isolate about twice as many exosomes as the Tim4-affinity or UC method, but a large amount of protein contamination, mainly from the FBS, was precipitated at the same time (as shown by Oriole staining for total protein detection) (Fig. 2b,c). In contrast, although the total yield of exosomes isolated by Tim4-affinity method was about half of that by the TEI method, protein contaminants were almost undetectable by Oriole staining. The UC method could isolate almost the same mass of exosomes as the Tim4-affinity method, but heavy protein contamination accompanied the exosomes when the centrifugation was done once without PBS wash. Addition of the second UC step after the PBS wash reduced contamination, but the yield of exosomes was also greatly diminished. To compare the purity of exosomes, the concentrations of total proteins in the isolated sEV fractions were determined by BCA protein assay, and the same amount of protein (100 ng) from each sample was subjected to SDS-PAGE. We confirmed that equal amounts of protein were loaded on the gel by Oriole staining. Western blot analysis against exosomal markers revealed much stronger enrichment of exosomal proteins such as CD63, CD9, and CD81 by the Tim4-affinity method than by the UC or TEI method (Fig. 2d,e). We found that only a very few residual exosomes in the flow through of 10K sup after Tim4-affinity purification could be isolated by UC, indicating that Tim4-affinity method have isolated most of the exosomes (Fig. 2f). We also compared the efficiency of exosomal purification by quantifying the yield of exosomal miRNAs and mRNAs^23^. Quantitative PCR analysis of miR-16, miR-92a, miR-142-3p, or GAPDH mRNA in the isolated sEV fractions revealed three to four less Ct values by the Tim4-affinity method than by the UC method indicating that about ten times more exosomal RNAs were isolated (Supplementary Fig. 1a,b). Taken together, these data suggest that Tim4-affinity is an ideal method to isolate sEVs containing exosomes with much higher purity than that by using other conventional methods. We also confirmed that the Tim4-affinity method could be used for the efficient isolation of sEVs from various cell lines and primary cells (Supplementary Fig. 2) as well as from biofluids such as serum and urine (Fig. 2g,h).

### Characterization by density gradient analysis

**Figure 3:**
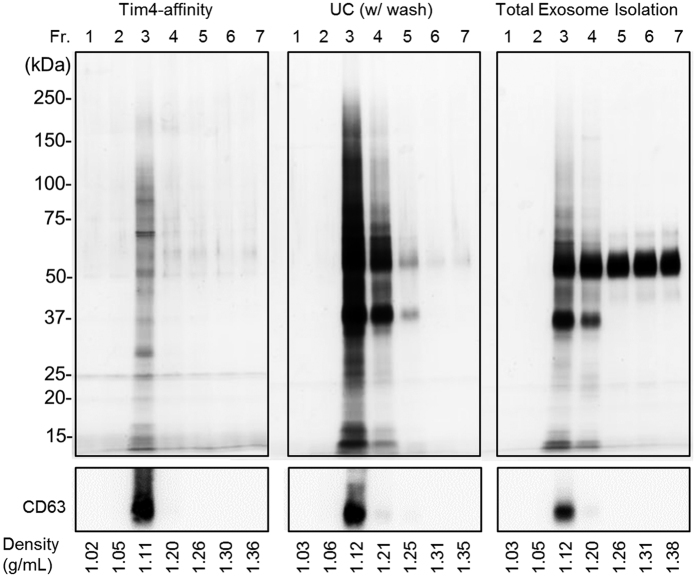
Density distribution of isolated sEVs. sEVs isolated from 10K sup of K562 cells by each method were subjected to density gradient fractionation analysis by using OptiPrep. After centrifugation, series of fractions were collected and measured for density. The fractionated samples were concentrated, lysed with SDS sample buffer, and separated by SDS-PAGE. Proteins in each fraction were visualized by silver staining (for total proteins, upper panels) or western blotting with an exosomal marker (CD63) (lower panels).

We next subjected the isolated sEVs to density gradient fractionation analysis (seven fractions) to compare enrichment of exosomes by each method, as exosomes typically float at density around 1.15 g/ml on sucrose gradient^24^. Indeed, western blot analysis against an exosomal marker (CD63) revealed the enrichment of exosomes in the third fraction, which corresponds to density of 1.11–1.12 g/ml by any purification method (Fig. 3). Then whole proteins in the each fractionated sample were visualized by silver staining. While most proteins isolated by the Tim4-affinity method were enriched in the third fraction, large amounts of proteins were also observed in the fourth and fifth fractions (density of 1.21–1.25 g/ml) by the UC method and in the fourth to seventh fractions (density of 1.20–1.38 g/ml) by the TEI method, which are not exosomal. These data indicate that sEV isolation by Tim4-affinity method can enrich exosomes more efficiently than other conventional methods.

### Proteomic analysis of sEVs

**Figure 4:**
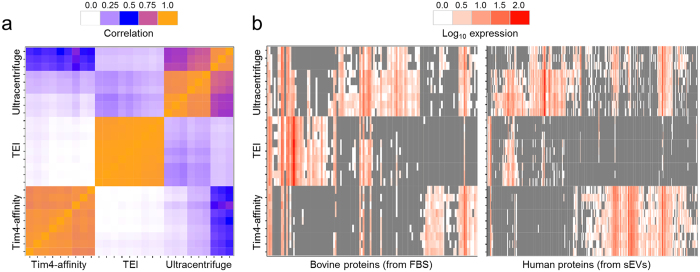
Comparison of protein components of sEVs. (**a**) Heatmap of pair-wise correlation between identified proteins in the sEV fractions isolated by each method. (**b**) Heatmap of expression levels for bovine (left panel) or human (right panel) proteins in the sEV fractions from K562 cells. The proteins were digested with trypsin and analyzed by mass spectrometry. The detected peptides were searched against Swiss-Prot using MASCOT, and identified proteins were compared. Proteins with a basal expression level are shown in grey, and highly expressed proteins are shown in red. The experiments were performed three times in biological triplicate each time for a total of nine replicates per sample.

To systematically assess the performance of our Tim4-affinity purification method against alternative methods, we used shotgun proteomics analysis to examine the entire complement of proteins extracted from sEV preparations isolated from 10K sup of K562 cells containing 10% FBS which had been deprived of any EVs and potential precipitates in advance. The protein profiles from experiments that used the Tim4-affinity or TEI methods showed high consistencies among biological and technical triplicates, whereas the UC method (with PBS wash) gave results with greater variation (Fig. 4a). Identified proteins were sorted into either bovine (non-sEV contaminants derived from FBS) or human (mostly sEV proteins derived from K562 cells) proteins, based on the species match from the MASCOT search. The abundance of each protein was estimated based on the number of peptides (Total Spectral Count, see methods). While the total number of peptides identified by mass spectrometry was comparable among the three methods, more human peptides were detected by the Tim4-affinity method (631.6 peptides, 78.1% of total peptides) than by the UC (608.3 peptides, 66.0% of total peptides) or TEI (109.3 peptides, 21.8% of total peptides) methods (Table 1). When the proteome of each sample was visualized as a heatmap, we found that Tim4-affinity purified sEV fractions tended to contain fewer identified bovine protein contaminants than those in sEV fractions from other methods, and the total abundance of the bovine contaminants was also lower (Fig. 4b). However, the number of different human proteins and their total abundance was higher than that by using other methods. This tendency was supported by examination of the top 30 proteins sorted by the number of peptides, which showed many more bovine protein contaminants in the sEV fractions purified with UC (12 proteins) or TEI (25 proteins), while only 5 proteins were found by the Tim4-affinity method (Supplementary Table 1). This suggests that sEVs extracted with the Tim4-affinity method have higher purity than those obtained using the UC or TEI method. Statistical analysis was performed to test whether there were significant differences in the concentrations of purified proteins obtained by the Tim4-affinity method compared to the UC or TEI method (Supplementary Table 2). This analysis confirmed that many bovine protein contaminants were lower, whereas many human proteins were significantly more abundant in Tim4-affinity purified sEV fractions. Most notably, many known human sEV proteins, such as several annexins (ANXA1-4, 6, 7, 11), flotillins (FLOT1 and 2), and syntenin (SDCB1) were among the most abundant recovered proteins, suggesting that the Tim4-affinity method enriches sEV proteins more efficiently than that by the UC or TEI method.

To examine whether exosomal proteins were enriched in Tim4-affinity purified sEV fractions, we performed functional enrichment analysis. Lists of proteins found in various types of exosomes were downloaded from the KEGG BRITE database (see methods) and enrichment analysis was performed against the list of Tim4-purified proteins (Table 2). The top two significantly enriched terms were “proteins found in most exosomes” and “proteins found in exosomes from hematopoietic cells,” supporting the conclusion that Tim4-affinity purified fractions were enriched in exosomal proteins. As the heatmap analyses revealed low correlations of protein profiles among the three methods (Fig. 4a,b), we next examined whether Tim4-affinity purified fractions were more enriched in exosomal proteins than that by the other purification methods (Supplementary Table 3). Again, proteins found in most exosomes and in hematopoietic exosomes were the top enriched terms, indicating that the Tim4-affinity purification method results in higher enrichment of exosome-specific proteins than that by the UC or TEI method. Taken together, these results indicate that the Tim4-affinity purification method increases the amount of exosomal proteins in the sEV fractions while reducing the amount of non-exosomal protein contaminants in comparison with the UC or TEI method.

### Quality comparison of sEVs and isolation of large EVs

**Figure 5:**
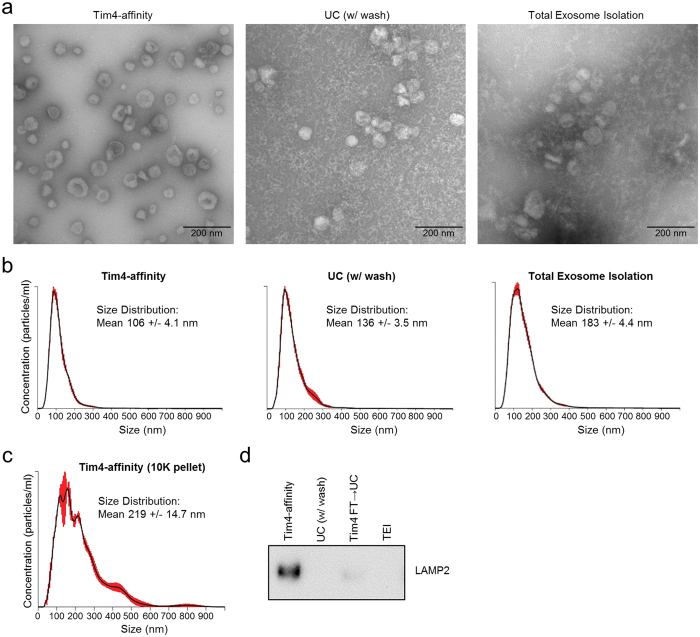
Evaluation of sEVs and large EVs. (**a**) sEVs (1 × 10^10^ particles) from 10K sup of K562 cells (serum free) isolated by each method were placed on a carbon-coated grid, and allowed to be absorbed onto the grid for 3 min. After being rinsed with distilled water, the sample was stained with a 2% aqueous uranyl acetate solution and examined with a JEOL JEM1011 electron microscope operating at 80 kV. Scale bar, 200 nm. (**b**) Isolated sEVs were diluted to a suitable concentration with PBS, and the size distribution of sEVs was analyzed by NTA using NanoSight LM10. Red error bars indicate +/−1 standard error of the mean. (**c**) 10K pellet of K562 cell conditioned medium was resuspended in 4 ml RPMI1640 supplemented with 2 mM CaCl_2_, and large EVs were isolated by Tim4-affinity method. The size distribution of the vesicles was analyzed by NTA. (**d**) Large EVs were isolated by each method from 10K pellet, and equal amount of proteins (100 ng) were subjected to SDS-PAGE, and analyzed by western blot against an EV marker (LAMP2). The flow through of 10K pellet after Tim4-affinity purification was further ultracentrifuged, and the same proportion of the protein amount to the total purified protein amount as Tim4-affinity purification was loaded to detect residual large EVs (Tim4 FT → UC).

We further compared the quality of sEVs isolated by the three methods. This time, we prepared sEVs from K562 cells by culturing the cells in serum-free medium to minimize the amount of protein contamination from FBS. First, the morphology of sEVs was examined by transmission electron microscope (TEM). The appearance of sEVs isolated by the Tim4-affinity method matched the typical saucer-like shape as previously reported^24^, and almost no contaminants could be observed (Fig. 5a). In contrast, sEVs isolated by UC (with PBS wash) and TEI methods were accompanied by a large number of small precipitates probably derived from supplements (such as human albumin, insulin, and transferrin) added in advance into the medium. Second, the size distribution of sEVs was examined by NTA using NanoSight. The mean size of sEVs isolated by either the UC or TEI method was larger (mean size: 136 nm or 183 nm in diameter, respectively) than that isolated by the Tim4-affinity method (mean size: 106 nm in diameter) due to increased populations of aggregated or fused sEVs larger than 200 nm (Fig. 5b). These data demonstrated that the Tim4-affinity method could isolate sEVs with higher quality than that by using conventional methods.

We next examine whether Tim4-affinity method could be also applicable to the purification of large EVs. As mentioned above, we pre-cleared K562 cell conditioned medium by sequential centrifugation at 300× *g*, 2,000× *g*, and 10,000× *g* and filtration to enrich sEVs in the 10K sup for purification. This time, we used 10K pellet, which was resuspended in RPMI1640 supplemented with 2 mM CaCl_2_, as a source of large EVs. We confirmed that mean size of the vesicles isolated by Tim4-affinity method from the 10K pellet was 219 nm in diameter, and large EVs up to 600 nm in diameter could be isolated by this method (Fig. 5c). We then compared the purity of large EVs isolated by the each method from the 10K pellet. When the same amount of protein (100 ng) from each purified products was subjected to SDS-PAGE and western blot against an EV marker (LAMP2), we found much stronger enrichment of LAMP2 by the Tim4-affinity method than that by the UC or TEI method (Fig. 5d). We also found that only a very few residual large EVs in the flow through after Tim4-affinity purification could be isolated by UC, indicating that Tim4-affinity method have isolated most of the large EVs. These data suggest that Tim4-affinity method is also an ideal method to isolate large EVs containing microvesicles with high purity from 10K pellet.

### ELISA and flow cytometry analyses of EVs

**Figure 6:**
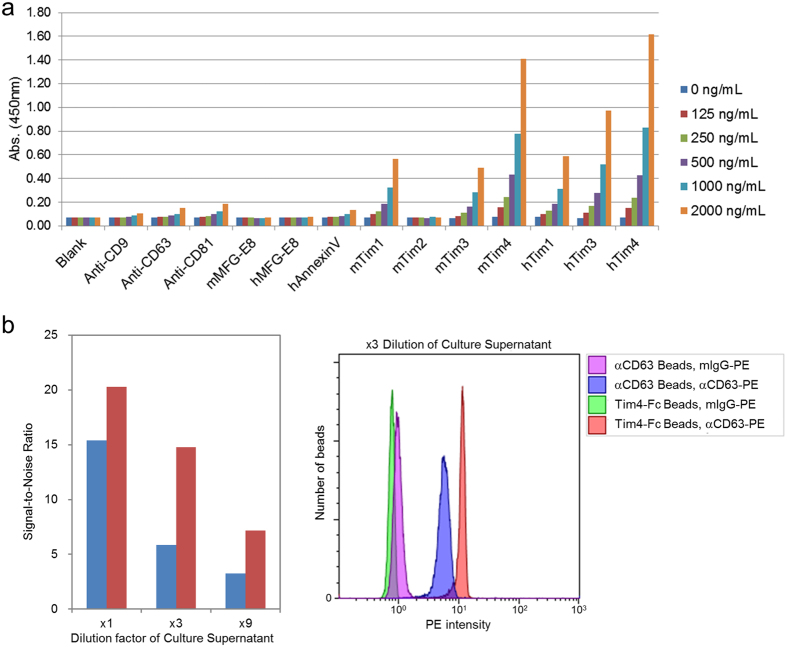
ELISA and FACS quantification of EVs. (**a**) sEVs in 10K sup of K562 cells (serum free) were purified by ultracentrifugation and the concentration of total proteins was determined by BCA protein assay. The sEVs were serially diluted and then incubated in each well of a Nunc MaxiSorp 96 well plate that had been pre-coated with one of the following capture reagents: anti-CD9, anti-CD63, anti-CD81 antibody, mouse MFG-E8-His, human MFG-E8-His, human AnnexinV-His, mouse Tim1-Fc, Tim2-Fc, Tim3-Fc, Tim4-Fc, human Tim1-Fc, Tim3-Fc, or Tim4-Fc. Bound exosomes were detected with HRP-conjugated anti-CD63 monoclonal antibody, followed by a chemiluminescence reaction with TMB solution. The absorbance at 450 nm was measured with Ultra Evolution plate reader (Tecan). (**b**) sEVs in 10K sup of K562 cells (serum free) were serially diluted with RPMI1640 supplemented with 2 mM CaCl_2_ (×1, ×3, ×9), and then captured with beads conjugated either with anti-CD63 mouse monoclonal antibody (blue bars) or mouse Tim4-Fc (red bars). Bound exosomes were labeled using either PE-conjugated anti-CD63 mouse monoclonal antibody or PE-conjugated Mouse IgG1 κ Isotype Control, and analyzed using flow cytometry. Signal to Noise ratios (signal intensities of anti-CD63-PE divided by those of anti-mouse IgG1-PE) are shown in the left graph and the FACS profiles of samples diluted 1:3 are shown in the right panel.

Quantifying the amount of EVs is essential for assuring quality control in the functional studies of EVs. NTA is one of the most commonly used methods to count the number of EV particles, but it requires an expensive apparatus. Because of the strong ability of Tim4 to capture EVs, we considered the possibility that it might be applied to the lower-cost sandwich ELISA technique. Proteins known to bind EVs (anti-CD9, CD63, or CD81 antibodies; MFG-E8, Annexin V, or Tim-Fc proteins) were coated on a microtiter plate, and various concentrations of sEVs (determined by total protein measurements) isolated from 10K sup of K562 cells by the UC method were allowed to be captured by them. Exosomes captured by these proteins were detected by anti-CD63 antibody. As expected, mouse and human Tim4-Fc captured exosomes much more efficiently than that by other proteins or antibodies, enabling a very sensitive and linear dose-dependent quantification of exosomes by ELISA (Fig. 6a). Likewise, the Tim4-affinity method could also be applied to analysis of EVs by flow cytometry. Beads conjugated either with Tim4-Fc or anti-CD63 antibody were incubated with 10K sup of K562 cells, and the bound exosomes were detected with PE-conjugated anti-CD63 antibody or its isotype control antibody. As expected, Tim4-conjugated beads captured exosomes more efficiently than that by CD63-conjugated beads, which led to a 2–3 fold stronger signal to noise ratio (Fig. 6b). These data demonstrated that the Tim4-affinity method could be used for sensitive and reliable quantification of EVs.

## Discussion

We have demonstrated the advantages of our Tim4-affinity method in the isolation of highly purified EVs over conventional methods such as UC and TEI. We found that this technique is particularly useful for the identification of EV proteins by a proteomics analysis because of the lower amount of non-EV protein contamination, which interferes with the analysis. This method will allow us to reveal EV components that have not been detectable by other methods and will help us to identify novel biomarkers in EVs. We also proved that Tim4 functions well in the ELISA and flow cytometry formats, and thus, can be utilized for sensitive and reliable quantification of EVs without the need for expensive NTA equipment.

As we described, this method is based on the high affinity of Tim4 protein for phosphatidylserine, which is displayed on the surface of EVs. Therefore, the Tim4-affinity method cannot distinguish between exosomes and microvesicles. Phosphatidylserine spontaneously translocates across the cell’s plasma membrane, but is normally restricted to the inner leaflet in healthy cells by the action of an ATP-dependent aminophospholipid flippases, which translocate phosphatidylserine from the outer to the inner leaflet of the plasma membrane^25^,^26^. When cells undergo apoptosis, these flippases are cleaved by caspases and this asymmetrical distribution is disrupted, allowing an exposure of phosphatidylserine on the cell surface^27^. Budding of EV leads to the separation of EVs from the cytosol, which contains mitochondria and ATP-generating machinery, resulting in exhaustion of ATP inside the EV and subsequent exposure of phosphatidylserine on the outer surface of the EV.

In the case of apoptotic events, exposed phosphatidylserine serves as an “eat me” signal for phagocytes^28^,^29^. Phosphatidylserine displayed on EVs is also recognized by phosphatidylserine receptors such as Tim4 that stimulate the internalization of EVs by the target cells^14^,^15^,^16^,^17^. Although the definition of “EVs” has been a matter of dispute, the use of *in vivo* receptors with natural affinity for EVs would seem to be a reasonable guide in defining a key physiological feature of functional EVs. Accordingly, typical exosomal proteins were much more enriched in sEVs isolated by the Tim4-affinity method. The UC and TEI methods are based on the principles of precipitation and sedimentation. As these methods non-specifically concentrate sedimentable particles, including non-sEV proteins, these separation techniques are not suitable for designating their products as rigorous sEVs. Moreover, as we observed in Fig. 4a, the UC method is associated with low reproducibility due to the loss of unstable and invisible pellets after centrifugation. Furthermore, the UC method has a variety of protocols, which may cause inconsistent results in different laboratories. On the other hand, the Tim4-affinity method is feasible, requiring only general lab equipment, yet it can easily isolate EVs with higher quality and reproducibility. Thus, the Tim4-affinity method is ideal for the isolation of EVs, and it will accelerate the studies of EV researchers.

## Methods

### Cell culture and preparation of cell culture supernatant

Any EVs and potential precipitates in FBS (Biowest) were depleted in advance by a combination of polymeric precipitation and ultracentrifugation. In brief, FBS and 1/5 volume of 50% (w/v) PEG 10,000 (Sigma-Aldrich) in PBS were mixed, and placed at 4 °C overnight, to allow precipitation of all EVs and precipitable proteins present. Then the mixture was centrifuged at 2,000× *g* for 30 min, and the supernatant was re-centrifuged at 100,000× *g* for 16 h using CS100FNX with S50ST swing rotor (Hitachi, Japan). K562 cells (mycoplasma negative) were obtained from RIKEN BRC and cultured in RPMI1640 (Nacalai Tesque, Japan) supplemented with 5% or 10% EV-depleted FBS or in X-VIVO serum-free medium (Lonza). K562 cells were induced to secrete EVs by the addition of 10 μM monensin sodium salt (Merck Millipore) and were incubated for 24 h post induction. The pMac were obtained 3 days after injection of 2 ml of 3% brewer thioglycollate medium (Sigma-Aldrich) into the peritoneal cavity of C57BL/6 mice (10-week-old female mice, SLC, Japan). pMac were cultured in DMEM (Nacalai Tesque, Japan) supplemented with 5% or 10% EV-depleted FBS for 48 h. The cell conditioned medium was collected and sequentially centrifuged at 300× *g* for 10 min, 2,000×*g* for 20 min, and 10,000× *g* for 30 min and filtered through 0.22 μm Millex-GV filter (Merck Millipore) to remove cells, cellular debris, and large EVs, resulting in the final pre-cleared cell culture supernatant (10K sup). Mice were housed in a pathogen-free facility at Osaka University and all animal experiments were carried out in accordance with relevant guidelines and regulations. All experimental protocols were approved by the Animal Research Committee of the Research Institute for Microbial Diseases at Osaka University.

### Ultracentrifugal and polymeric precipitation of sEVs

For isolation of sEVs by ultracentrifugation, 10K sup was centrifuged at 100,000× *g* for 2 h using CS100FNX with S50ST swing rotor. The supernatant was discarded by pipetting, and the pellet was resuspended with PBS (UC w/o wash). The mixture was re-centrifuged at 100,000× *g* for 2 h and the pellet was resuspended with PBS (UC w/wash). For polymeric precipitation, 10K sup was mixed with 0.5 volume of Total Exosome Isolation Reagent (Thermo Fisher Scientific) and incubated overnight at 4 °C. The mixture was centrifuged at 10,000× *g* for 1 h and the pellet was resuspended with PBS.

### Tim4-affinity purification of sEVs

To isolate EVs by the Tim4-affinity method, MagCapture Exosome Isolation Kit PS (Wako, Japan) was used according to the manufacturer’s instructions. In brief, 0.6 mg of streptavidin magnetic beads, bound with 1 μg of biotinylated mouse Tim4-Fc, was added to 10K sup supplemented with 2 mM CaCl_2_ and the mixture was rotated overnight at 4 °C. The beads were washed three times with 1 ml of washing buffer (20 mM Tris-HCl, pH 7.4, 150 mM NaCl, 0.0005% Tween20, 2 mM CaCl_2_), and the bound sEVs were eluted with elution buffer (20 mM Tris-HCl, pH 7.4, 150 mM NaCl, 2 mM EDTA).

### Gel electrophoresis and western blotting

Purified EV fractions were lysed with 2× SDS sample buffer (100 mM Tris-HCl, pH 6.8, 4% (w/v) SDS, 20% (v/v) glycerol) and separated by SDS-PAGE. Total proteins were visualized with Oriole Fluorescent Gel Stain (Bio-Rad). Western blot signal was detected and analyzed by using Luminata Western Chemiluminescent HRP Substrates (Merck Millipore) and ImageQuant LAS 4000mini (GE Healthcare). The following antibodies were obtained from BioLegend: LEAF Purified anti-human CD63 (H5C6, 1:1,000), Purified anti-mouse CD63 (NVG-2, 1:1,000), Purified anti-human CD81 (5A6, 1:1,000), Purified anti-mouse/rat CD81 (Eat-2, 1:1,000), Purified anti-human CD9 (HI9a, 1:1,000), Purified anti-mouse CD9 (MZ3, 1:1,000), and Purified anti-human LAMP2 (H4B4, 1:1,000). Purified Mouse Anti-Flotillin-2 (29/Flotillin-2, 1:2,000) was from BD Biosciences.

### Density gradient centrifugation

Isolated sEVs were dissolved in 1.8 ml of 47% OptiPrep (Axis-Shield) in 20 mM HEPES, pH7.4, and loaded in ultracentrifugation tubes. The samples were overlaid with four successive layers (2 ml each) of 20 mM HEPES containing 47%, 37%, 28% and 18% OptiPrep. The tubes were ultracentrifuged at 200,000× *g* for 16 h at 4 °C. After centrifugation, series of fractions (1.4 ml each × 7) were collected, diluted with 2.8 ml of 20 mM HEPES, and concentrated to 100 μl with VivaSpin 6-3K (GE Healthcare). The fractionated samples were lysed with SDS sample buffer and separated by SDS-PAGE using a SuperSep Ace 5–20% polyacrylamide gel (Wako). Proteins were visualized by silver staining using a silver staining kit (Wako) or western blotting against CD63.

### Mass spectrometry and statistical analysis

sEVs were purified from 10K sup of K562 cells using the Tim4, UC, or TEI method, in biological triplicates. Purified sEV samples were loaded on a 10% polyacrylamide gel and entire protein bands were excised. Peptides were quantified by LC-ESI-MS/MSQ-TOF Ultima (Waters, Japan) in technical triplicates per biological replicate, and proteins were identified by MASCOT search. Protein abundances were quantified using Scaffold software (version 3.4.5) and Total Spectrum Count (TSC) values were determined. Only proteins mapping to cow (*Bos bovis*) and human were considered for further analysis and contaminating human keratin proteins were disregarded. All statistical analyses were performed with the R software for statistical analysis (http: www.r-project.org) and packages from the Bioconductor project^30^. Consistency between technical and biological replicate samples was assessed using the Pearson correlation distance (*d* = 1−*ρ*(*x, y*), where *ρ* is the Pearson correlation coefficient between the protein profiles of samples *x* and *y*). The correlation distance matrix was visualized with a heatmap with rows and columns representing samples and grouped using a hierarchical clustering algorithm (complete linkage) implemented in the *hclust* function available in R. To determine sEV purity, the average TSC among biological and technical triplicates was calculated for each protein and purification method. Then, the ratio between the amount of human and human plus cow proteins was calculated. For statistical analysis, protein levels for technical replicates were averaged using the *avereps* function in the *limma* package^31^. Differences in protein abundances measured as TSCs were used to assess the performance of the different purification methods. To determine differentially purified proteins we used linear regression models implemented in the *limma* package. The model included the different purification methods as predictors, and the comparisons Tim4 vs. UC and Tim4 vs. TEI were computed. Proteins were considered differentially purified at a false discovery rate (FDR) of <1% 32. Functional enrichment analysis was performed to identify over-representation of proteins found in exosomes. For this, a list of proteins found in different types of exosomes was downloaded from the KEGG BRITE database (http://www.genome.jp/kegg-bin/get_htext?ko04147.keg), and over-representation was assessed using a hypergeometric test implemented in the R function *phyper*. Two types of enrichment tests were performed. In one, we selected all proteins with TSC>1 in Tim4 to test whether the purified exosomal proteins could have been obtained by chance. In the other, we selected proteins increased in Tim4-derived samples from the list of proteins differentially purified by Tim4 compared to the other methods, to test whether Tim4 purification tends to purify more exosomal proteins than that by TEI or UC. In all cases enrichment was considered significant at an FDR of <1%.

### EV ELISA

Antibodies used as capture reagents: anti-CD9 (M-L13), anti-CD63 (H5C6), anti-CD81 (JS-81) mouse monoclonal antibodies (all from BD Bioscience). Proteins used as capture reagents: mouse and human MFG-E8-His (R&D Systems), human AnnexinV-His (Creative BioMart), mouse Tim2-His (R&D Systems), mouse Tim1-Fc, Tim3-Fc, Tim4-Fc, human Tim1-Fc, Tim3-Fc, and Tim4-Fc (all from Wako, Japan). The above antibodies and proteins were immobilized to the wells of a Nunc MaxiSorp 96 well plate (Thermo Fisher Scientific) at a concentration of 1 μg/well in 50 mM MOPS (pH 7.5) for 16 h at 4 °C. Non-specific binding sites were blocked with 25% Block Ace solution (DS Pharma Biomedical, Japan). After removing blocking solution, 100 μl of ultracentrifuged sEV fraction (protein concentration: 125–2000 ng/ml) dissolved in reaction buffer (20 mM Tris-HCl, pH 7.4, 150 mM NaCl, 2 mM CaCl_2_) was added to each well and incubated for 1 h at room temperature. Then, each well was washed three times with 300 μl of washing buffer (20 mM Tris-HCl, pH 7.4, 150 mM NaCl, 0.05% Tween20, 2 mM CaCl_2_). The bound exosomes were quantified by ELISA using HRP-labeled anti-CD63 mouse monoclonal antibody (HRP was attached using peroxidase labeling kit-NH_2_, Dojindo) and TMB solution (Wako, Japan). After stopping the reaction with 1 M HCl, the absorbance at 450 nm was measured with Ultra Evolution plate reader (Tecan).

### EV Flow cytometry

Anti-CD63 mouse monoclonal antibody and mouse Tim4-Fc were labeled with biotin using biotin labeling kit-SH (Dojindo, Japan). 2 × 10^5^ beads of EV-Streptavidin Isolation/Detection Reagent (Thermo Fisher Scientific) bound either with 0.1 μg of biotinylated anti-CD63 mouse monoclonal antibody or biotinylated mouse Tim4-Fc was added to 100 μl of 10K sup of K562 cells (serum free) supplemented with 2 mM CaCl_2_, and mixed by rotation overnight at 4 °C. The beads were washed twice with 300 μl washing buffer (20 mM Tris-HCl, pH 7.4, 150 mM NaCl, 0.0005% Tween20, 2 mM CaCl_2_), and resuspended with the washing buffer. Exosomes bound to the beads were labeled using either PE-conjugated anti-CD63 mouse monoclonal antibody (H5C6, BD Bioscience) or PE-conjugated Mouse IgG1 κ Isotype Control (BD Bioscience) for 1 h at room temperature and then analyzed using Beckman Coulter Gallios and Kaluza software (Beckman Coulter).

## Additional Information

**How to cite this article**: Nakai, W. *et al*. A novel affinity-based method for the isolation of highly purified extracellular vesicles. *Sci. Rep.* **6**, 33935; doi: 10.1038/srep33935 (2016).

## Supplementary Material

Supplementary Information

Supplementary Information

## Figures and Tables

**Table 1:** sEV purity calculated as the average ratio of human proteins over total proteins (human plus bovine).

Method	Total	Human	Ratio
Tim4	808.7	631.6	78.1%
UC	921.3	608.3	66.0%
TEI	501.9	109.3	21.8%

Each value for protein abundance is the average number of peptides from nine replicates (biological and technical triplicates).

**Table 2:** Enrichment analysis using proteins purified by Tim4-affinity method (TSC>1).

KEGG BRITE category	Ntotal	Nset	P_value_	FDR
Proteins found in most exosomes	88	16	0	0
Exosomal proteins of hematopoietic cells	281	17	0	0
Exosomal proteins of other body fluids	354	13	2.60E-12	7.81E-11
Exosomal proteins of colorectal cancer cells	283	10	7.34E-10	2.13E-08
Exosomal proteins of bladder cancer cells	139	7	7.71E-09	2.16E-07
Exosomal proteins of hepatic cells	26	3	9.15E-07	2.47E-05
Exosomal proteins of breast cancer cells	30	2	8.79E-05	2.29E-03
Exosomal proteins of other cancer cells	40	2	2.09E-04	5.24E-03

Ntotal: number of proteins in category; Nset: number of Tim4-affinity purified proteins in category; FDR: false discovery rate.
