# Supplementary material for: A novel affinity-based method for the isolation of highly purified extracellular vesicles
Source: Sci Rep. 2016 Sep 23;6:33935. doi: 10.1038/srep33935 (PMC5034288; doi:10.1038/srep33935)
Supplement: Supplementary Information [file srep33935-s1.doc]

**A novel affinity-based method for the isolation of highly purified extracellular vesicles**

Wataru Nakai^1,6^, Takeshi Yoshida^1,3,6^, Diego Diez^2^, Yuji Miyatake^1,3^, Takahiro Nishibu^4^, Naoko Imawaka^4^, Ken Naruse^4^, Yoshifusa Sadamura^4^ & *Rikinari Hanayama^1,3,5^

^1^Laboratory of Immune Network, ^2^Quantitative Immunology Research Unit, WPI Immunology Frontier Research Center (IFReC), Osaka University, 3-1 Yamada-oka, Suita, Osaka 565-0871, Japan

^3^Department of Immunology, Kanazawa University Graduate School of Medical Sciences, 13-1 Takara, Kanazawa, Ishikawa 920-8640, Japan

^4^Life Science Research Laboratories, Wako Pure Chemical Industries Ltd., 6-1 Takada-Cho, Amagasaki, Hyogo 661-0963, Japan

^5^PRESTO, Japan Science and Technology Agency (JST), 4-1-8 Honcho, Kawaguchi, Saitama 332-0012, Japan

TEL: +81-76-265-2725 FAX: +81-76-265-2734

^6^These authors contributed equally to this work

*Correspondence and requests for materials should be addressed to R.H.

(email: rikinari-hanayama@umin.ac.jp).

**
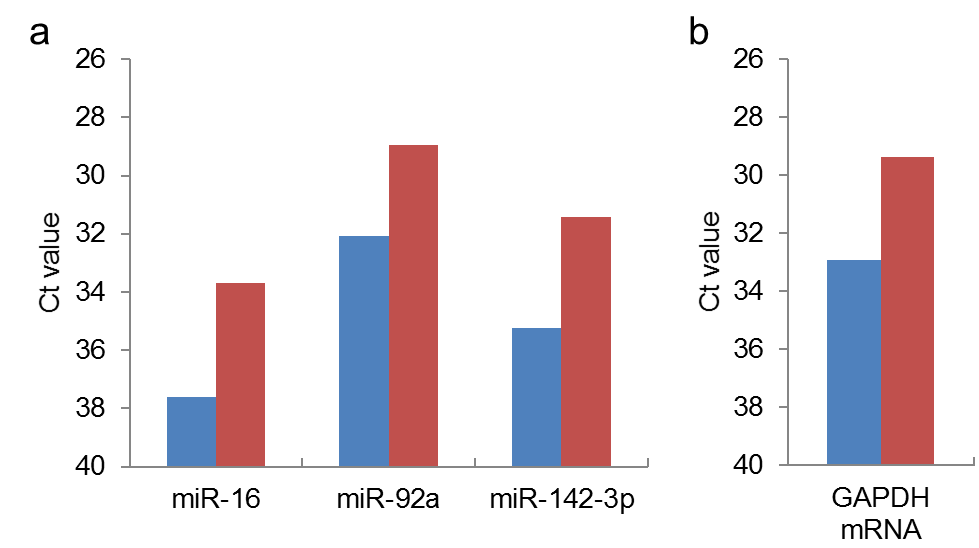
**

**Supplementary Figure 1** | **qPCR analysis of exosomal RNAs in sEV fractions purified by each method.** RNAs were extracted from sEVs isolated from 10K sup of K562 cells by Tim4-affinity (red bars) or UC (blue bars) method. (a) Individual miRNAs were reverse transcribed to cDNA using Taqman microRNA reverse transcription kit and stem-loop primers (Thermo Fisher Scientific). Quantitative PCR of miRNA was performed with Taqman microRNA assay kit and Taqman universal PCR master mix on an ABI 7500 real-time PCR system. (b) mRNAs were reverse transcribed to cDNA using SuperScript VILO cDNA synthesis kit (Thermo Fisher Scientific). Quantitative PCR of mRNA was performed with specific primers for GAPDH (forward: 5’-AGCCACATCGCTCAGACACC-3’, reverse: 5’-GTACTCAGCGCCAGCATCGC-3’) and Power SYBR Green PCR master mix.

**
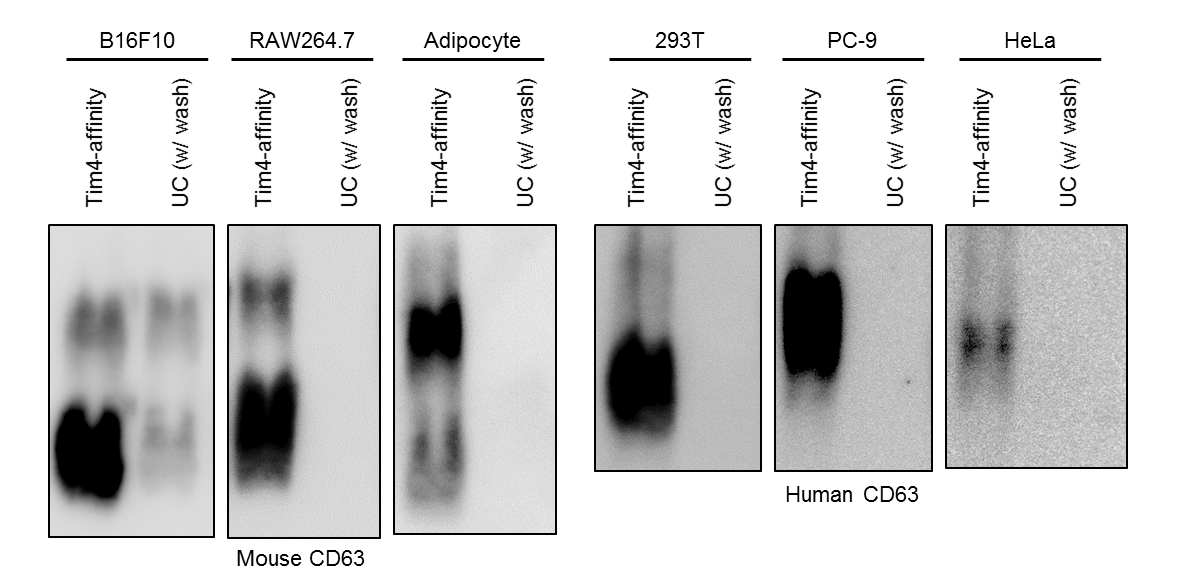
**

**Supplementary Figure 2** | **Purification of sEVs from various cell lines and primary cells.** sEVs were purified from 10K sup of each cells by Tim4-affinity or UC method. Protein concentration of purified sEV fractions was determined and equal amount of proteins (100 ng) were subjected to SDS-PAGE, and analyzed by western blot against mouse (left three panels) or human (right three panels) CD63. Cell lines and primary cells used in these experiments are as follows: B16F10 (mouse skin melanoma), RAW264.7 (mouse macrophage), mouse primary adipocyte, 293T (human embryonic kidney cell), PC-9 (human lung adenocarcinoma), HeLa (human cervical adenocarcinoma).

**Supplementary Table 1** | List of the 30 most abundant proteins detected in the sEV fractions from K562 cell culture supernatants by mass spectrometry, which was sorted by the average number of peptides. Gray columns indicate bovine protein contaminants derived from FBS.

| **Tim4** | | **UC** | | **TEI** | |
| --- | --- | --- | --- | --- | --- |
| **Identified Proteins** | **Mean** | **Identified Proteins** | **Mean** | **Identified Proteins** | **Mean** |
| Heat shock cognate 71 kDa protein | 26.1 | DNA-dependent protein kinase catalytic subunit | 53.7 | Complement C3 | 92.0 |
| Annexin A6 | 26.0 | Transferrin receptor protein 1 | 22.3 | Alpha-2-macroglobulin | 30.8 |
| Transferrin receptor protein 1 | 24.6 | Serum albumin | 20.4 | Fibronectin | 24.0 |
| V-type proton ATPase catalytic subunit A | 19.3 | ATP-dependent RNA helicase A | 20.4 | Serum albumin | 23.2 |
| Flotillin-2 | 18.7 | Tubulin beta-5 chain | 17.6 | Thrombospondin-1 | 19.3 |
| Programmed cell death 6-interacting protein | 18.7 | Heat shock cognate 71 kDa protein | 17.0 | Complement C4 | 18.2 |
| 4F2 cell-surface antigen heavy chain | 18.3 | Fatty acid synthase | 16.9 | Alpha-1-antiproteinase | 16.3 |
| Annexin A1 | 18.2 | 4F2 cell-surface antigen heavy chain | 16.8 | Apolipoprotein B-100 | 14.4 |
| Kinase D-interacting substrate of 220 kDa | 16.8 | U5 small nuclear ribonucleoprotein helicase | 15.3 | Hemoglobin fetal subunit beta | 12.1 |
| Annexin A2 | 15.0 | Tubulin beta-4B chain | 15.2 | Tubulin beta-5 chain | 9.9 |
| Flotillin-1 | 14.1 | Heterogeneous nuclear ribonucleoprotein M | 13.0 | Fatty acid synthase | 9.4 |
| V-type proton ATPase subunit B, brain isoform | 14.0 | Hemoglobin fetal subunit beta | 12.1 | Adiponectin | 9.1 |
| Annexin A11 | 13.4 | Clathrin heavy chain 1 | 12.0 | Fibulin-1 | 9.1 |
| Annexin A7 | 12.9 | Fibronectin | 11.0 | Complement C4-A | 8.8 |
| Heat shock 70 kDa protein 1A | 12.4 | Tubulin alpha-1B chain | 9.8 | Complement component C7 | 8.4 |
| Syntenin-1 | 12.4 | Alpha-1-antiproteinase | 9.0 | Apolipoprotein A-I | 8.0 |
| Nucleolin | 12.1 | Elongation factor 1-alpha 1 | 8.8 | Complement factor H | 8.0 |
| Na^+^/K^+^-transporting ATPase subunit alpha-1 | 11.8 | V-type proton ATPase subunit B, brain isoform | 8.6 | T-complex protein 1 subunit theta | 7.7 |
| Annexin A4 | 11.6 | Neutral amino acid transporter B(0) | 8.0 | Apolipoprotein E | 7.6 |
| Neutral amino acid transporter B(0) | 11.4 | Heterogeneous nuclear ribonucleoprotein U | 7.9 | Tubulin alpha-1B chain | 7.6 |
| Annexin A3 | 10.2 | Programmed cell death 6-interacting protein | 7.9 | Elongation factor 1-alpha 1 | 7.0 |
| Complement C4 | 10.0 | Histone H2B type 1-B | 7.8 | Heat shock protein HSP 90-alpha | 6.7 |
| ATP-binding cassette sub-family B member 6 | 9.1 | Histone H2B type 1-C/E/F/G/I | 7.8 | Gelsolin | 6.0 |
| Apolipoprotein B-100 | 9.1 | V-type proton ATPase catalytic subunit A | 7.7 | Tubulin beta-4B chain | 6.0 |
| Apolipoprotein E | 9.0 | Pre-mRNA-processing-splicing factor 8 | 7.6 | Alpha-2-HS-glycoprotein | 5.6 |
| Secretory carrier-associated membrane protein 3 | 8.1 | T-complex protein 1 subunit theta | 7.4 | Pigment epithelium-derived factor | 5.0 |
| Tubulin beta-5 chain | 7.2 | Kinase D-interacting substrate of 220 kDa | 7.3 | Antithrombin-III | 4.6 |
| Serum albumin | 7.1 | Nucleolar RNA helicase 2 | 7.1 | Inter-alpha-trypsin inhibitor heavy chain H3 | 4.6 |
| Erythrocyte band 7 integral membrane protein | 7.1 | Heat shock 70 kDa protein 1A | 6.9 | Complement factor B | 4.3 |
| Solute carrier family 2 | 7.0 | Eukaryotic initiation factor 4A-I | 6.7 | Collectin-43 | 4.1 |

**Supplementary Table 2** | Statistical analysis of proteins detected in the sEV fractions, comparing Tim4-affinity vs. UC or Tim4-affinity vs. the TEI method (shown in a separate Excel file).

**Supplementary Table 3** | Enrichment analysis using proteins differentially purified by Tim4-affinity vs. UC or Tim4-affinity vs. the TEI method. Ntotal: number of proteins in category; Nset: number of Tim4 purified proteins in category; FDR: false discovery rate.

Tim4 vs. UC

| **category** | **Ntotal** | **Nset** | **P_value_** | **FDR** |
| --- | --- | --- | --- | --- |
| Proteins found in most exosomes | 88 | 12 | 0 | 0 |
| Exosomal proteins of hematopoietic cells | 281 | 10 | 5.46E-12 | 1.69E-10 |
| Exosomal proteins of other body fluids | 354 | 10 | 6.46E-11 | 1.94E-09 |
| Exosomal proteins of hepatic cells | 26 | 3 | 1.54E-07 | 4.48E-06 |
| Exosomal proteins of bladder cancer cells | 139 | 5 | 2.42E-07 | 6.78E-06 |
| Exosomal proteins of colorectal cancer cells | 283 | 6 | 1.01E-06 | 2.72E-05 |
| Exosomal proteins of other cancer cells | 40 | 2 | 5.62E-05 | 1.46E-03 |
| Exosomal proteins of microglial cells | 15 | 1 | 3.44E-04 | 8.59E-03 |

Tim4 vs. TEI

| **category** | **Ntotal** | **Nset** | **P_value_** | **FDR** |
| --- | --- | --- | --- | --- |
| Proteins found in most exosomes | 88 | 12 | 0 | 0 |
| Exosomal proteins of hematopoietic cells | 281 | 14 | 2.22E-16 | 6.88E-15 |
| Exosomal proteins of other body fluids | 354 | 12 | 3.42E-12 | 1.03E-10 |
| Exosomal proteins of colorectal cancer cells | 283 | 7 | 4.12E-07 | 1.19E-05 |
| Exosomal proteins of bladder cancer cells | 139 | 5 | 1.06E-06 | 2.96E-05 |
| Exosomal proteins of hepatic cells | 26 | 2 | 3.19E-05 | 8.60E-04 |
